# Interleukin 4 promotes the development of ex-Foxp3 Th2 cells during immunity to intestinal helminths

**DOI:** 10.1084/jem.20161104

**Published:** 2017-06-05

**Authors:** Victoria S. Pelly, Stephanie M. Coomes, Yashaswini Kannan, Manolis Gialitakis, Lewis J. Entwistle, Jimena Perez-Lloret, Stephanie Czieso, Isobel S. Okoye, Dominik Rückerl, Judith E. Allen, Frank Brombacher, Mark S. Wilson

**Affiliations:** 1 Allergy and Anti-Helminth Immunity Laboratory, Mill Hill Laboratory, The Francis Crick Institute, London NW7 1AA, England, UK; 2 Ahr Immunity Laboratory, Mill Hill Laboratory, The Francis Crick Institute, London NW7 1AA, England, UK; 3 Faculty of Life Sciences (3IR), University of Manchester, Manchester M13 9PT, England, UK; 4 International Centre for Genetic Engineering and Biotechnology, University of Cape Town, Institute of Infectious Disease and Molecular Medicine and South African Medical Research Council, 7925 Cape Town, South Africa

## Abstract

Immunity to intestinal helminth infections requires the rapid activation of T helper 2 cells (Th2 cells). However, simultaneous expansion of CD4^+^Foxp3^+^ regulatory T cells (T reg cells) impedes protective responses, resulting in chronic infections. The ratio between T reg and effector T cells can therefore determine the outcome of infection. The redifferentiation of T reg cells into Th cells has been identified in hyperinflammatory diseases. In this study, we asked whether ex–T reg Th2 cells develop and contribute to type-2 immunity. Using multigene reporter and fate-reporter systems, we demonstrate that a significant proportion of Th2 cells derive from Foxp3^+^ cells after *Heligmosomoides polygyrus* infection and airway allergy. Ex-Foxp3 Th2 cells exhibit characteristic Th2 effector functions and provide immunity to *H. polygyrus*. Through selective deletion of *Il4ra* on Foxp3^+^ cells, we further demonstrate IL-4 is required for the development of ex-Foxp3 Th2 cells. Collectively, our findings indicate that converting T reg cells into Th2 cells could concomitantly enhance Th2 cells and limit T reg cell–mediated suppression.

## Abstract (precis)

Pelly et al. use novel mouse reporter systems to show that a proportion of Th2 cells develop from Foxp3-expressing cells in an IL-4–dependent manner, highlighting the potential to subvert T reg cell–mediated suppression in favor of type 2 immunity.

## Introduction

A heterogeneous population of regulatory T cells (T reg cells) is required to maintain immune homeostasis and limit excessive immune responses to infection (Belkaid, 2007; Campbell and Koch, 2011). However, protection from immune-mediated pathology and autoimmunity can also permit the establishment of chronic infections (Gause et al., 2013). Indeed, after a primary infection with the natural mouse parasite *Heligmosomoides polygyrus*, the early expansion (Grainger et al., 2010) and activation (Finney et al., 2007) of *Foxp3*-expressing T reg cells limits excessive T helper 2 cell (Th2 cell) responses and immunopathology, resulting in the establishment of chronic infections (Rausch et al., 2009). CD4^+^ T cells and the cytokine IL-4 are essential for the initiation of protective type-2 inflammatory mechanisms after *H. polygyrus* infection (Urban et al., 1991a,b). Th2 cell–derived IL-4, IL-5, and IL-13 orchestrate an effective wave of immune cell and tissue responses, including the activation of macrophages (Anthony et al., 2006), class switching of B cells (Wojciechowski et al., 2009; Esser-von Bieren et al., 2013), and promoting of the secretion of Relmβ from epithelial cells (Herbert et al., 2009). Th2 cells are also required for vaccination-mediated immunity to *H. polygyrus* (Hewitson et al., 2015), placing Th2 effector cells as an integral population of immune cells for both natural and vaccine-mediated immunity. It has been proposed that shifting the ratio of T reg and Th2 cells could improve immunity. Indeed, the adoptive transfer of effector CD4^+^ T cells from immune mice conferred immunity to susceptible hosts (Rausch et al., 2008), and conversely, T reg cell depletion resulted in increased type-2 responses (Rausch et al., 2009). Whether similar shifts in T reg and effector T cell populations occur in mice resistant to *H. polygyrus* is unclear.

Studies using fate-reporter systems have identified that in Th1/Th17-mediated autoimmune and inflammatory diseases, including models of rheumatoid arthritis (Komatsu et al., 2014), experimental autoimmune encephalomyelitis (Bailey-Bucktrout et al., 2013), and type-1 diabetes (Zhou et al., 2009), a proportion of Th cells originate from *Foxp3*-expressing cells. Whether such redifferentiation of Foxp3^+^ cells occurs during immunity to infection or during Th2 cell–mediated responses is unclear. Several lines of evidence suggest that T reg cells and Th2 cells may be closely related. Indeed, reduced levels of *Foxp3* in mouse (Wan and Flavell, 2007) and human (Hansmann et al., 2012) T cells or loss of cofactors required for the maintenance or function of T reg cells (Sawant et al., 2012; Jin et al., 2013; Muto et al., 2013; Roychoudhuri et al., 2013; Ulges et al., 2015) resulted in the acquisition of a Th2 cell phenotype. Furthermore, evidence from mouse and human cells identified that T reg cells from individuals suffering from oral allergy have a Th2 cell–like phenotype (Noval Rivas et al., 2015). In this study, we investigated whether T reg cells contributed to a protective Th2 memory response after infection with *H. polygyrus*. As expected, protective immunity correlated with an increase in Th2 cell frequencies and a reduction in Foxp3^+^ T reg cells. Fate reporter and adoptive transfer strategies identified that a significant proportion of Th2 cells originated from *Foxp3*-expressing cells after *H. polygyrus* infection or house dust mite (HDM)–induced airway allergy. Functionally, ex-Foxp3 Th2 cells could activate innate cells and provide immunity to *H. polygyrus*. In vitro and in vivo experiments found that selective deletion of IL-4Rα on Foxp3^+^ cells prevented the conversion of Foxp3^+^ cells to Th2 cells after *H. polygyrus* infection, demonstrating that IL-4 critically drives Th2 cell differentiation from both naive T cells (nT cells) and Foxp3^+^ T cells. Therapeutically converting T reg cells into Th2 cells may therefore bolster Th2 cell–mediated antihelminth immunity, providing both an extra source of effector Th2 cells and concomitantly reducing T reg cell frequencies.

## Results

### A shift from a regulatory to a polarized type-2 immune response during immunity to *H. polygyrus*

**Figure 1.**
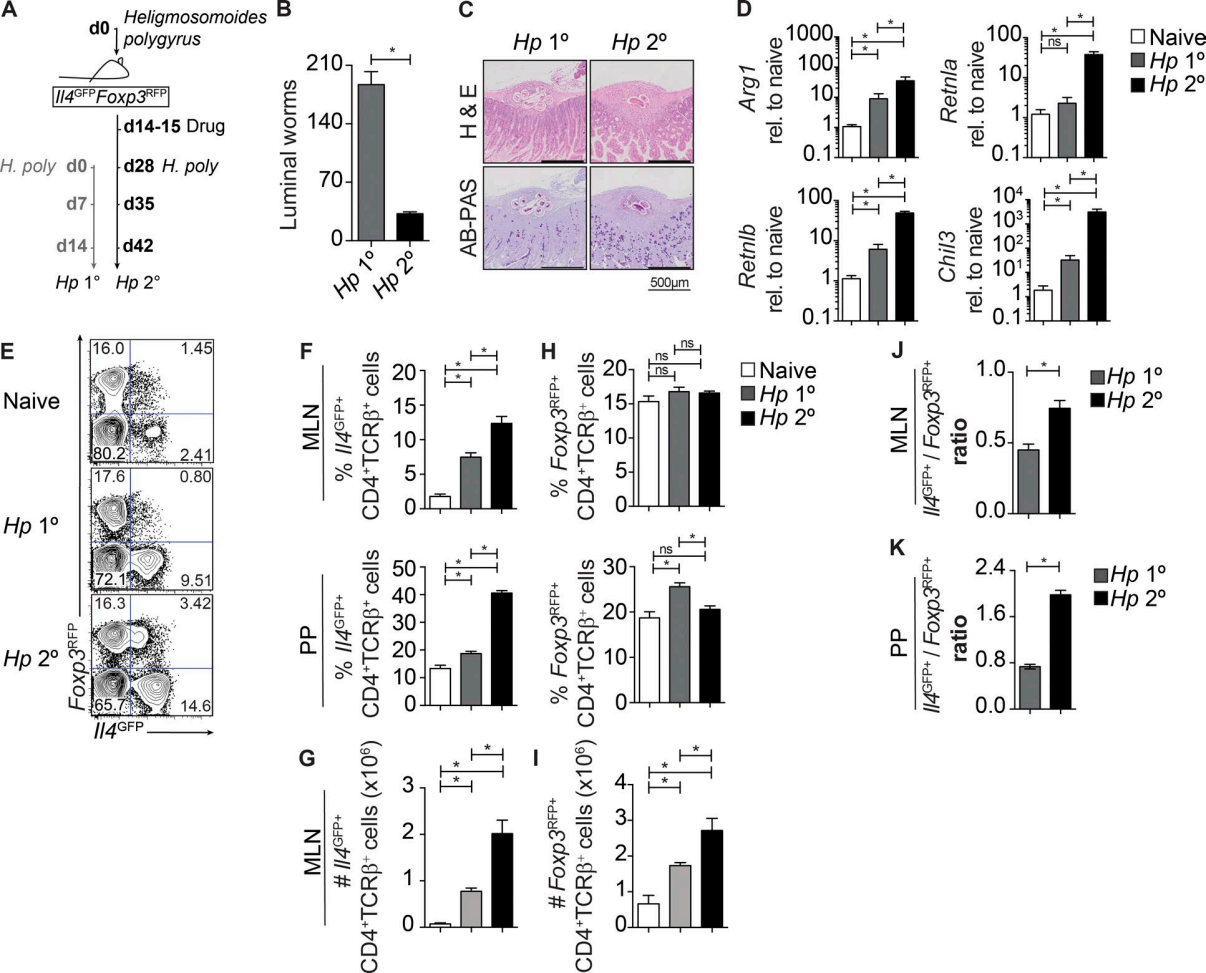
**A shift in the ratio of T reg to Th2 cells correlates with the functional expulsion of *H. polygyrus*.** (A) Experimental model. Female *Il4^GFP^Foxp3^RFP^* mice were infected with 200 *H. polygyrus* larvae. *Hp* 2° mice were treated with pyrantel embonate on days 14–15 and reinfected on day 28. *Hp* 1° mice were given a primary infection at the same time point. (B) Intestinal worm burden at day 14 after infection. Data are representative of at least three experiments with three mice per group. (C) Hematoxylin and eosin (H&E)– and Alcian blue–periodic acid–Schiff (AB-PAS)–stained sections of the small intestine of *Hp* 1° and *Hp* 2° mice day 7 after infection, depicting *H. polygyrus* larvae. (D) Gene expression of *Arg1*, *Retnla*, *Retnlb*, and *Chil3* in the small intestine of *Hp* 1° and *Hp* 2° mice day 7 after infection, expressed as fold-change relative (rel.) to naive small intestine. Data represent six to seven mice per group pooled from two independent experiments. (E) Representative FACS plots of *Il4^GFP^* and *Foxp3^RFP^* expression in CD4^+^TCRβ^+^ MLN cells of naive, *Hp* 1°, and *Hp* 2° mice day 7 after infection. (F–I) Proportion (F) and absolute number (G) of *Il4^GFP+^* cells and *Foxp3^RFP+^* cells (H and I) in the MLN and PPs of naive, *Hp* 1°, and *Hp* 2° mice day 7 after infection. (J and K) Ratio of *Il4^GFP+^* cells over and *Foxp3^RFP+^* cells in the MLN (J) and PPs (K). Data are representative of three experiments with three to five mice per group. *, P ≤ 0.05; one-way ANOVA or Mann-Whitney test. Error bars represent SEM.

Intestinal helminths establish chronic infections in mammalian hosts because of the development of inappropriate immune responses. Similarly to their human hookworm counterparts, primary infections with the natural mouse helminth *H. polygyrus* (*Hp* 1°) result in a chronic infection (Fig. 1, A and B). However after the secondary infection of drug-cured immune mice (*Hp* 2°), invading *H. polygyrus* larvae are killed in the tissue, resulting in reduced numbers of adult worms emerging into the lumen (Fig. 1, A and B). Distinct immune pathways have been shown to be involved in immunity to *H. polygyrus*, such as increased intestinal inflammation (Fig. 1 C, H&E), mucus (Fig. 1 C, AB-PAS), and goblet cell–derived Relmβ (*Retnlb*) secretion and the alternative activation of macrophages (AAMΦ; *Arg1*, *Retnla*, and *Chil3*; Fig. 1 D; Urban et al., 1991a; Anthony et al., 2006; Herbert et al., 2009). CD4^+^ Th2 cells orchestrate much of this type-2 immune response. However, the ontogeny of Th2 effector cells during protective immunity is unclear. Furthermore, whereas T reg cells expand and limit Th2 effector cells during primary infections with *H. polygyrus* (Finney et al., 2007; Rausch et al., 2009; Grainger et al., 2010), the involvement of T reg cells during protective immunity is unclear.

We generated a dual-reporter mouse (*Il4^GFP^Foxp3^RFP^*) by crossing *Il4*- (4get) and *Foxp3*-reporter (*FILIG*) mice (Mohrs et al., 2001; Wan and Flavell, 2007) to accurately monitor *Il4* and *Foxp3*–expressing T cells. Using this mouse, we simultaneously analyzed the dynamics of CD4^+^TCRβ^+^*Il4^GFP+^* (Th2) and CD4^+^TCRβ^+^*Foxp3^RFP+^* (T reg) cells in *Hp* 1° and *Hp* 2° mice (Fig. 1 E). The proportion of Th2 cells in the mesenteric LNs (MLN) and Peyer’s patches (PPs; Fig. 1 F) and absolute number of Th2 cells in the MLN (Fig. 1 G) of *Hp* 1° mice were significantly increased. Furthermore, *Hp* 2° mice had even greater Th2 cells in the MLN and PPs, compared with naive or *Hp* 1° mice. In contrast, the proportion of T reg cells increased in the PPs of *Hp* 1° mice but not in *Hp* 2° mice and were maintained in the MLN (Fig. 1 H). Consequently, despite an increase in the absolute number of T reg cells (Fig. 1 I), there was a significant increase in the ratio of Th2 cells over T reg cells in the MLN and PPs of *Hp* 2° mice (Fig. 1, J and K), correlating with immunity to *H. polygyrus*.

### Foxp3^+^CD25^high^ T reg cells convert to Th2 cells after adoptive transfer into *H. polygyrus*–infected T cell–deficient mice

**Figure 2.**
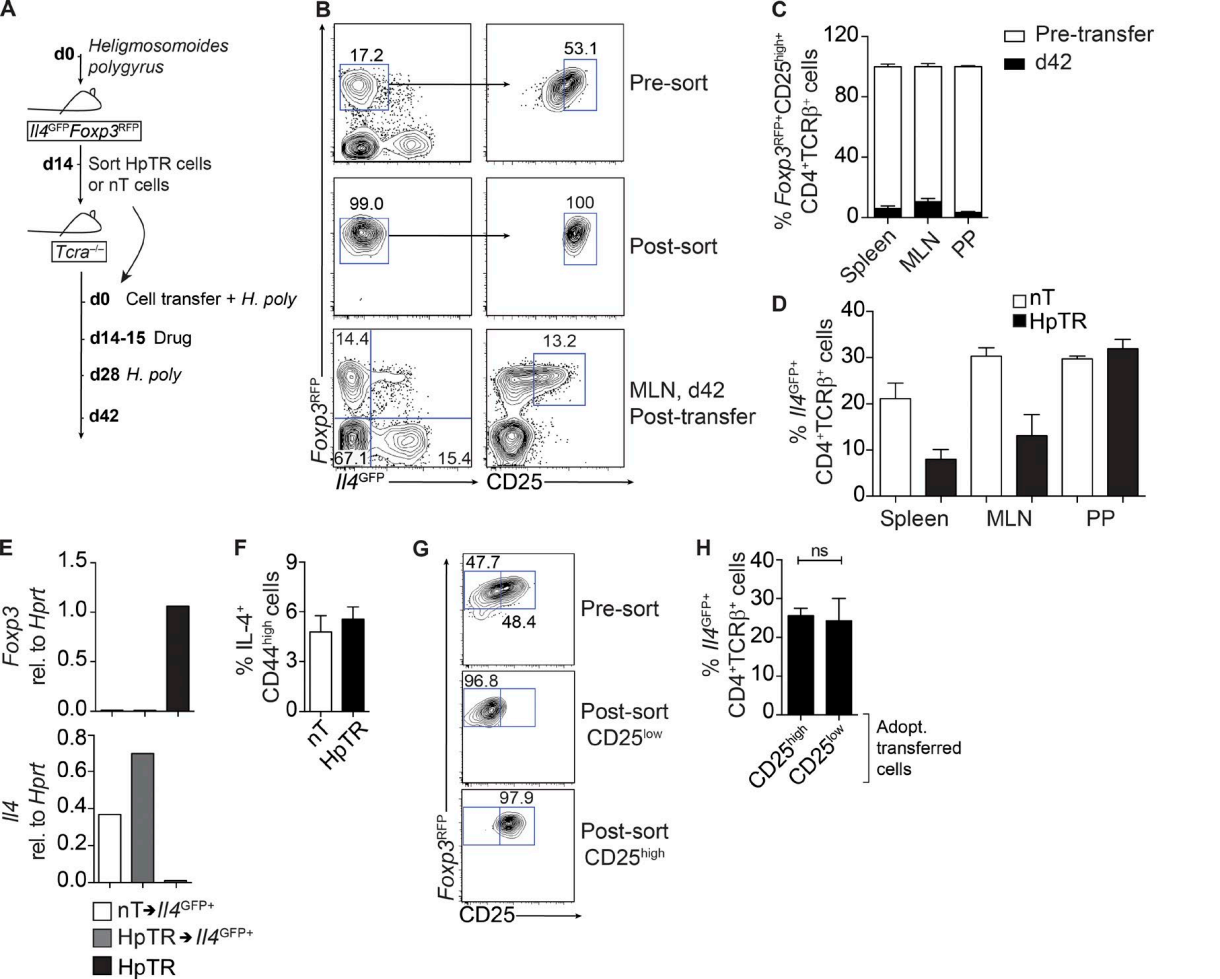
**Adoptively transferred T reg cells lose *Foxp3* expression and convert to *Il4*-expressing cells after secondary infection with *H. polygyrus*.** (A) Experimental model. T reg cells (HpTR; CD4^+^TCRβ^+^*Il4^GFP–^Foxp3^RFP+^*CD25^high^) were sort purified from male *Il4^GFP^Foxp3^RFP^* mice infected with *H. polygyrus* for 14 d. nT cells (CD4^+^TCRβ^+^CD25^–^CD44^low^*Il4^GFP–^Foxp3^RFP–^*) were sort purified from the spleen and MLN of naive male *Il4^GFP^Foxp3^RFP^* mice. HpTR or nT cells were transferred to male *Tcra^−/−^* mice further subjected to a secondary infection with *H. polygyrus*. (B) Representative FACS plots of *Foxp3^RFP^*, *Il4^GFP^*, and CD25 expression in HpTR cells before and after sort and day 42 after transfer. (C and D) Proportion of *Foxp3^RFP+^*CD25^high^ cells in HpTR recipients day 42 after transfer and relative to *Foxp3^RFP^* expression before transfer (C) and proportion of CD4^+^TCRβ^+^*Il4^GFP+^Foxp3^RFP–^* cells in the spleen, MLN, and PPs of nT or HpTR cell recipients day 42 after transfer (D). Data are representative of at least five independent experiments with three to five mice per group. (E) qRT-PCR validation of *Foxp3* and *Il4* expression relative (rel.) to *Hprt* in sort-purified nT→*Il4^GFP+^*, HpTR→*Il4^GFP+^*, or HpTR cells. Data are representative of three experiments with cells pooled from three to five recipients. (F) Frequency of CD4^+^CD44^high^IL-4^+^ cells in the MLN of nT and HpTR cell recipients as measured by intracellular cytokine staining. Data are representative of three independent experiments with four mice per group. CD4^+^TCRβ^+^*Foxp3^RFP+^*CD25^high^ or CD25^low^ cells were sort purified from *Il4^GFP^Foxp3^RFP^* mice infected with *H. polygyrus* for 14 d and transferred to *Tcra^−/−^* mice subjected to a secondary infection with *H. polygyrus*. (G) Representative FACS plots of *Foxp3^RFP^* and CD25 expression in donor HpTR cells presort. (H) Proportion of CD4^+^TCRβ^+^*Il4^GFP+^* cells in the PPs of *Hp* 2° recipients day 42 after transfer. Data are representative of two independent experiments with four to five mice per group. Adopt., adoptively. Error bars represent SEM.

After the observation that there was a shift in the ratio of Th2 cells to T reg cells during protective immunity, coupled with evidence of phenotypic plasticity between T cell populations (Barbi et al., 2014), we hypothesized that T reg cells may contribute to, rather than regulate, the Th2 cell pool during protective immunity. To determine whether T reg cells could be redifferentiated to become Th2 effector cells, we established an adoptive transfer model into lymphodeficient hosts (Fig. 2 A) as a proof of principle to study T reg conversion, similar to that used in the original study demonstrating T reg cell instability and acquisition of effector properties (Duarte et al., 2009). We purified T reg cells from *H. polygyrus*–infected donor mice (HpTR; CD4^+^TCRβ^+^*Il4^GFP−^Foxp3^RFP+^*CD25^high^) or nT cells (CD4^+^TCRβ^+^*Il4^GFP–^Foxp3^RFP^*^–^CD44^low^CD25^–^) from naive mice and adoptively transferred them into T cell–deficient mice. Recipient mice were subjected to a secondary *H. polygyrus* infection (Fig. 2 A). After transfer, ∼80% of transferred HpTR cells (which were ∼100% *Foxp3^RFP+^*CD25^high^ upon transfer) had lost *Foxp3* expression and down-regulated CD25 (Fig. 2, B and C), with 10–20% of the ex-Foxp3 cells in the spleen, MLN, and PP expressing *Il4^GFP^* (Fig. 2, B and D). We purified T reg cells that had lost *Foxp3* expression and up-regulated *Il4* (HpTR→*Il4^GFP+^*) and confirmed reduced expression of *Foxp3* and elevated *Il4* expression by quantitative real-time PCR (qRT-PCR; Fig. 2 E). HpTR→*Il4^GFP+^* cells also secreted IL-4 after restimulation (Fig. 2 F). Previous studies have identified that high expression of CD25 correlated with the functional stability of T reg cells in vitro (Komatsu et al., 2009) and in vivo after adoptive transfer (Miyao et al., 2012). However, the development of HpTR→*Il4^GFP+^* cells after *H. polygyrus* infection occurred independently of the levels of CD25 expression, with similar conversion observed after the transfer of CD25^low^ or CD25^high^ Foxp3^+^ cells from *H. polygyrus*–infected mice (Fig. 2, G and H).

**Figure 3.**
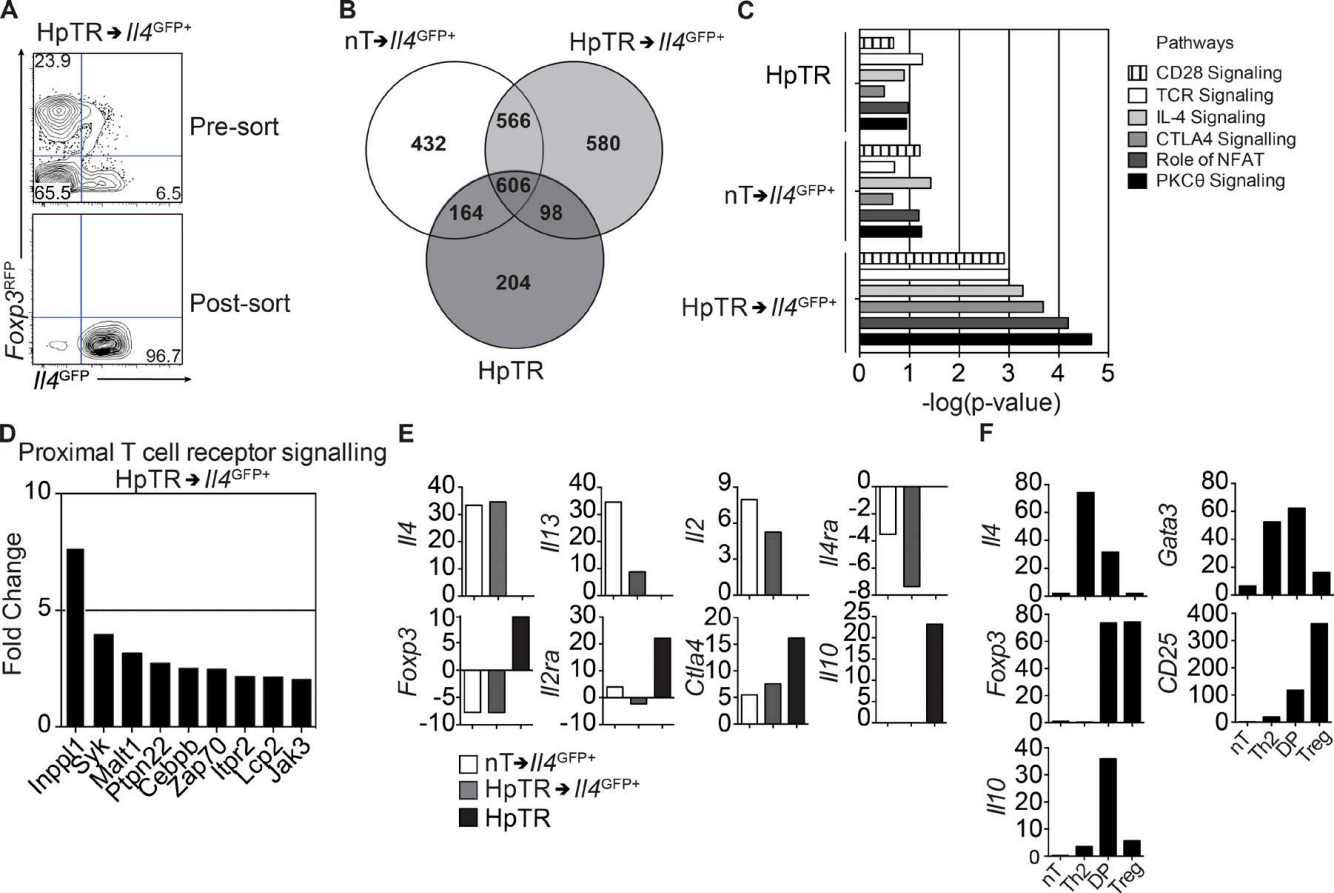
**Gene expression profiling reveals ex-Foxp3 cells express characteristic Th2 cell–related genes.** (A) Representative FACS plots of *Il4^GFP^* and *Foxp3^RFP^* expression in sort-purified *Il4^GFP+^* cells originating from transferred HpTR cells (HpTR→*Il4^GFP+^*). Gene expression profiling was performed using a microarray. (B) Venn diagram showing comparative analysis of common and uniquely expressed genes relative to nT cells between donor HpTR cells, HpTR→*Il4^GFP+^* cells, and *Il4^GFP+^* cells originating from transferred nT cells (nT→*Il4^GFP+^*). (C) Pathway analyses of differentially expressed genes in HpTR→*Il4^GFP+^* cells compared with nT→*Il4^GFP+^* and HpTR cells. For each biological replicate, cells were sorted from three to four mice. This figure represents a mean gene expression for three biological replicates (D) Genes involved in proximal TCR signaling, uniquely expressed in HpTR→*Il4^GFP+^* cells. (E) Expression of Th2 and T reg cell–related genes in HpTR cells, HpTR→*Il4^GFP+^*, or nT→*Il4^GFP+^* relative to nT cells. Data are representative of one experiment with three biological replicates per group. For each biological replicate, cells were sorted from three to four mice. T reg cells (HpTR; CD4^+^TCRβ^+^*Il4^GFP–^Foxp3^RFP+^*CD25^high^), Th2 (CD4^+^TCRβ^+^*Il4^GFP+^Foxp3^RFP–^*) cells, and double-positive cells (CD4^+^TCRβ^+^*Il4^GFP+^Foxp3^RFP+^*) were sort purified from male *Il4^GFP^Foxp3^RFP^* mice infected with *H. polygyrus* for 14 d. nT cells (CD4^+^TCRβ^+^CD25^–^CD44^low^*Il4^GFP–^Foxp3^RFP–^*) were sort purified from the spleen and MLN of naive male *Il4^GFP^Foxp3^RFP^* mice. (F) Expression of Th2 and T reg cell–related genes, relative to *Hprt* as assessed by qRT-PCR. Data are representative of two experiments with cells pooled from four to six donor mice in each experiment. DP, double positive.

To determine how transcriptionally similar converted HpTR→*Il4^GFP+^* were to conventional Th2 cells (nT cells that had up-regulated *Il4^GFP^* [nT→*Il4^GFP+^*]), we sort purified *Il4^GFP+^* cells from *Tcra^−/−^* mice that had received nT or HpTR cells (Fig. 3 A) and determined their global gene expression by microarray. HpTR→*Il4^GFP+^* were transcriptionally much more similar to nT→*Il4^GFP+^* cells than their T reg cell past, with 1,172 (566 + 606) transcripts in common between HpTR→*Il4^GFP+^* and nT→*Il4^GFP+^*, compared with 704 (98 + 606) genes in common with their ancestor T reg cell lineage (Fig. 3 B; see Table S1 for list of genes). Pathway analyses of differentially expressed genes in HpTR→*Il4^GFP+^* cells identified elevated PKCθ, NFAT, CTLA4, IL-4, TCR, and CD28 signaling, compared with Th2 (nT→*Il4^GFP+^*) and HpTR cells (Fig. 3 C; see Table S2 for list of genes). HpTR→*Il4^GFP+^* cells also had 580 unique genes that were differentially regulated, including several genes involved in proximal TCR signaling (Fig. 3 D; see Table S1 for list of genes). Thus, HpTR→*Il4^GFP+^* cells had significantly rewired their transcriptional profile, with ∼64% of the differentially expressed genes similar to nT→*Il4^GFP+^* cells (Fig. 3 B). In particular, HpTR→*Il4^GFP+^* cells expressed hallmark Th2 cell–associated genes such as *Il4*, *Il13*, and *Il2* and had down-regulated *Il4ra* expression (Fig. 3 E), as previously shown after Th2 cell differentiation in vivo (Perona-Wright et al., 2010). In addition, HpTR→*Il4^GFP+^* cells down-regulated the expression of T reg cell–associated genes such as *Il2ra*, *Ctla4*, and *Il10* as well as *Foxp3*, compared with HpTR cells (Fig. 3 E). Together, these data identified that *Foxp3^RFP+^*CD25^high^ T reg cells have the potential to convert to IL-4–expressing and –secreting cells, similar to nT cells.

### Ex-Foxp3 Th2 cells develop naturally and participate in secondary immune responses to *H. polygyrus*

**Figure 4.**
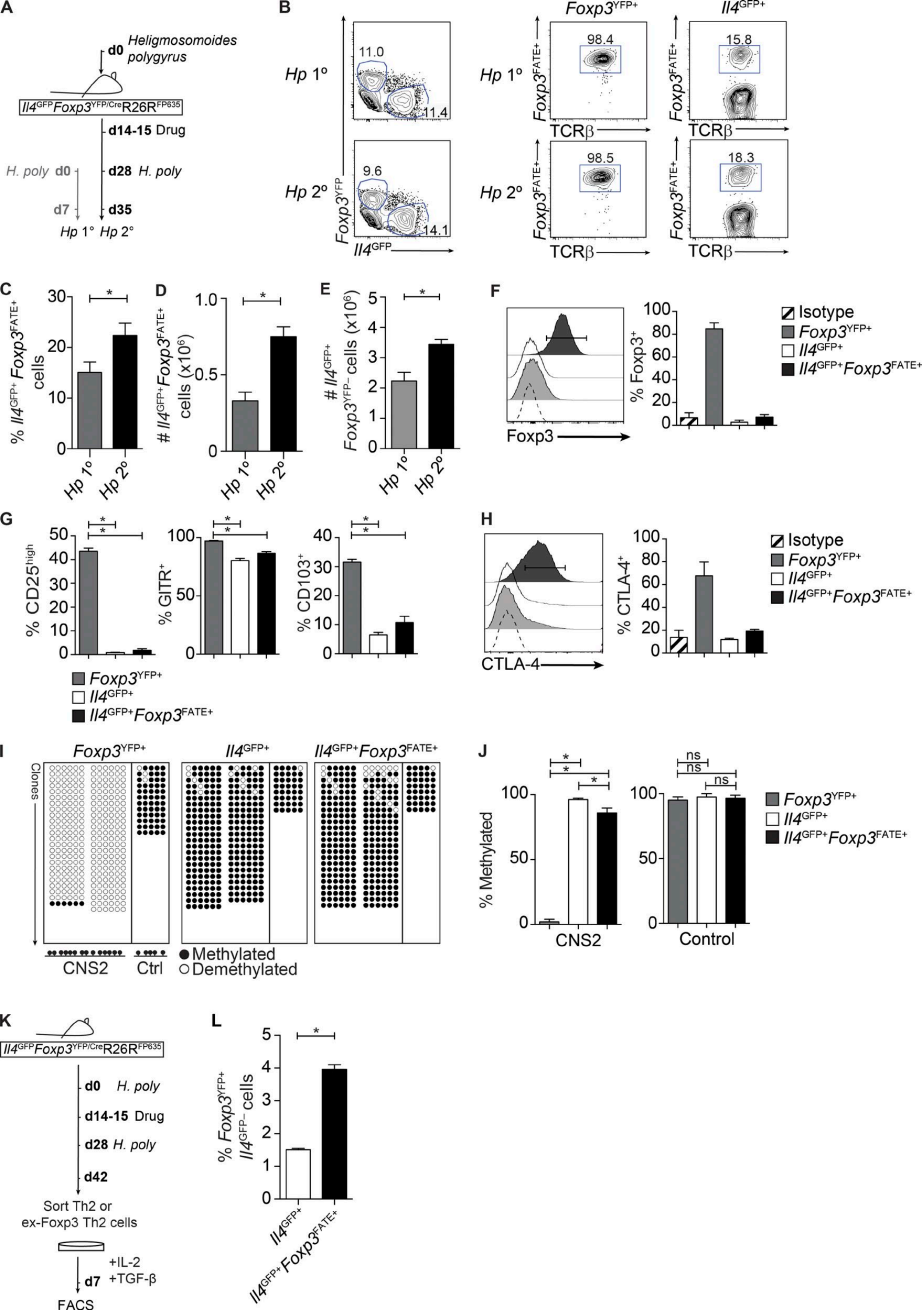
**A proportion of Th2 cells originate from a *Foxp3*-expressing past and expand after secondary infection with *H. polygyrus*.** (A) Experimental model. Female *Il4^GFP^Foxp3^YFP/Cre^*R26R^FP635^ mice were infected with 200 *H. polygyrus* larvae. *Hp* 2° mice were treated with pyrantel embonate on day 14–15 and reinfected on day 28. *Hp* 1° mice were given a primary infection at the same time point. (B) Representative FACS plots of FP635 expression (*Foxp3^FATE^*) within CD4^+^TCRβ^+^*Il4^GFP–^Foxp3^YFP+^* (*Foxp3^YFP+^*) and CD4^+^TCRβ^+^*Il4^GFP+^Foxp3^YFP–^* (*Il4^GFP+^*) cells. (C–E) Proportion (C) and absolute number (D) of CD4^+^TCRβ^+^*Il4^GFP+^Foxp3^FATE+^* cells and absolute number of total CD4^+^TCRβ^+^*Il4^GFP+^* Th2 cells (E) in the spleen of *Hp* 1° and *Hp* 2° mice day 7 after infection. Data represent 8–14 mice/group pooled from three independent experiments. (F) Intracellular Foxp3 protein expression in sort-purified T reg (*Foxp3^YFP+^Foxp3^FATE+^*), Th2 (*Il4^GFP+^Foxp3^YFP–^Foxp3^FATE–^*), and ex-Foxp3 Th2 (*Il4^GFP+^Foxp3^YFP–^Foxp3^FATE+^*) cells. Two mice per group were used. (G and H) CD25, GITR, CD103 (G), and intracellular CTLA-4 (H) protein expression in sort-purified T reg, Th2, and ex-Foxp3 Th2 cells. Data represent two to three independent experiments (with two to five mice per experiment). Bisulfite modification of DNA was performed followed by the amplification, cloning, and sequencing of the T reg cell–specific demethylated region of the *Foxp3* locus (CNS2). (I and J) Pictorial (I) and graphical (J) representation of the frequency of methylated cytosines in the *Foxp3* CNS2 and control (Ctrl) region of sort-purified T reg, Th2, and ex-Foxp3 Th2 cells. Data are pooled from two independent experiments with five mice per experiment and represent 8–27 clones per region. (K) *Il4^GFP+^* or *Il4^GFP+^Foxp3^FATE+^* cells were sort purified from *Il4ra^wt/wt^* or *Il4ra^fl/fl^ Hp* 2° fate-reporter mice day 7 after infection and cultured in T reg cell–polarizing conditions for 7 d. (L) Proportion of *Foxp3^YFP+^* cells at day 7. Data are representative of two independent experiments. Sorted T cells were pooled from four to five mice per experiment. *, P ≤ 0.05; Mann-Whitney test or one-way ANOVA. Error bars represent SEM.

To determine whether the differentiation of IL-4–expressing cells from *Foxp3^RFP+^*CD25^high^ T reg cells occurred in lympho-complete environments, we generated a fate-reporter system by crossing *Foxp3^YFP/Cre^* (Rubtsov et al., 2008) and *R26R^FP635^* (Coomes et al., 2015) mice on an *Il4^GFP^* background (Mohrs et al., 2001) to generate *Il4^GFP^Foxp3^YFP/Cre^R26R^FP635^* mice, allowing us to identify whether *Il4^GFP+^* Th2 cells originated from *Foxp3^YFP+^* cells (*Il4^GFP+^Foxp3^FATE+^*) during *Hp* 1° and *Hp* 2° infection (Fig. 4, A and B, left). As expected, the majority (∼98%) of cells expressing *Foxp3^YFP^* were marked with FP635 (*Foxp3^FATE^*; Fig. 4 B, middle; Miyao et al., 2012), indicating that they were currently (*Foxp3^YFP+^*) and had in the past (*Foxp3^FATE+^*) expressed *Foxp3*. Strikingly, during secondary infection with *H. polygyrus*, a substantial proportion (up to 20%) of *Il4^GFP+^* Th2 cells were *Foxp3^YFP−^Foxp3^FATE+^* (Fig. 4 B, right), with significantly higher proportions in *Hp* 2° relative to *Hp* 1° mice (Fig. 4 C). Furthermore, the absolute number of ex-Foxp3 Th2 cells (*Il4^GFP+^Foxp3^FATE+^Foxp3^YFP−^*) were significantly increased in *Hp* 2° mice (Fig. 4 D), contributing to the significant increase in absolute number of Th2 cells overall (Fig. 4 E). These data identify that a significant proportion of Th2 cells derive from *Foxp3*-expressing cells after *H. polygyrus* infection, with increased ex-Foxp3 Th2 cells correlating with immunity.

### Ex-Foxp3 Th2 cells lose characteristics of their T reg cell past

As expected, ex-Foxp3 Th2 cells had largely lost Foxp3 protein expression, in accordance with the reporter expression (Fig. 4 F). Furthermore, ex-Foxp3 Th2 cells expressed reduced levels of CD25, glucocorticoid-induced TNFR-related protein (GITR), CD103, and CTLA-4, compared with *Foxp3^YFP+^* cells and were similar to Th2 cells (Fig. 4, G and H). Commitment toward the T reg cell lineage requires the establishment of a unique DNA methylation landscape on the *Foxp3* locus and other T reg cell–associated genes, termed T reg cell–specific demethylation regions. Three conserved noncoding regions (CNS1, CNS2, and CNS3) are primary targets of DNA methylation in the *Foxp3* locus and play important roles in T reg cell development, function, and stability (Zheng et al., 2010). In particular, hypomethylation of CNS2 is required for the maintenance of T reg cell stability and function in the periphery (Zheng et al., 2010; Feng et al., 2014). In contrast to HpTR cells whose *Foxp3* locus was largely demethylated (Fig. 4, I and J), we found that a high proportion of ex-Foxp3 Th2 cells displayed a methylated *Foxp3* locus (Fig. 4, I and J), similar to Th2 cells. A small but significant proportion of ex-Foxp3 Th2 cells displayed a demethylated *Foxp3* locus, highlighting some degree of heterogeneity in the ex-Foxp3 Th2 population (Fig. 4 J) and suggesting that a small proportion of ex-Foxp3 Th2 cells may be able to reexpress *Foxp3*. Indeed, ex-Foxp3 Th2 cells had the capacity to reexpress *Foxp3^YFP^* after culture under T reg cell–polarizing conditions in vitro (Fig. 4, K and L). However, this was only a minor population. Together, these data suggest that ex-Foxp3 Th2 cells had largely lost characteristic elements of their *Foxp3*-expressing past and, instead, contribute to the Th2 effector response during infection with *H. polygyrus*, particularly during proficient immunity.

### IL-4 promotes T reg to Th2 cell conversion in vitro

**Figure 5.**
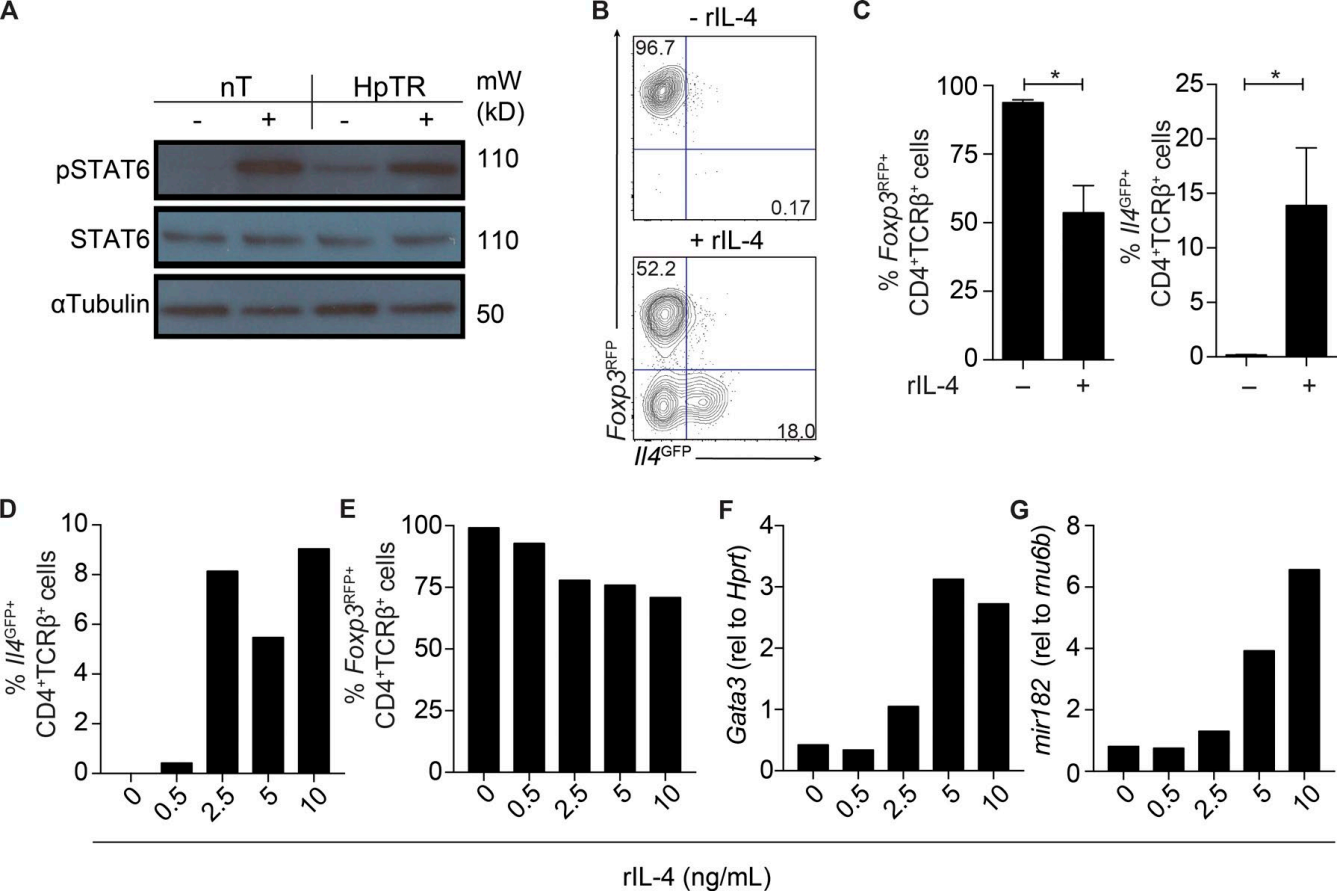
**IL-4 is sufficient to promote T reg to Th2 cell conversion in vitro.** (A–G) HpTR cells and nT cells were sort purified from *Il4^GFP^Foxp3^RFP^* reporter mice as described in Fig. 2. Sorted cells were stimulated with recombinant IL-4 at 37°C for 15 min or with media as a control. (A) Levels of pSTAT6, total STAT6, and α-tubulin protein in restimulated cells. Data are representative of three independent experiments. Sorted T cells were pooled from three to four mice. mW, molecular weight. (B and C) nT or HpTR cells were cultured with anti-CD3/CD28 and IL-2, with and without the addition of IL-4. Representative FACS plots (B) and graph (C) showing the frequency of CD4^+^TCRβ^+^*Foxp3^RFP+^* and CD4^+^TCRβ^+^*Il4^GFP+^* cells in day 7 cultures are shown. HpTR cells were cultured with anti-CD3/CD28 and IL-2, with increasing concentrations of IL-4, and cells were harvested for FACS or qRT-PCR at day 7. (D and E) Frequency of CD4^+^TCRβ^+^*Il4^GFP+^* (D) and CD4^+^TCRβ^+^*Foxp3^RFP+^* (E) cells in day 7 cultures. (F and G) Gene expression of *Gata3* (relative [rel] to *Hprt*; F) and *mir182* (relative to *rnu6b*; G) in cultured cells at day 7. Data are representative of two independent experiments. Cells were sort purified from four to six mice. *, P ≤ 0.05; Mann-Whitney test. Error bars represent SEM.

IL-4Rα signaling via STAT6 leads to the differentiation of conventional Th2 effector cells (Kaplan et al., 1996; Takeda et al., 1996). To determine whether IL-4 could promote T reg cell redifferentiation into Th2 cells, we exposed purified HpTR cells to IL-4. After 15 min, STAT6 in ex-Foxp3 Th2 cells was phosphorylated to similar levels as in nT cells exposed to IL-4 (Fig. 5 A). After 7 d of culture, a large proportion of HpTR cells lost *Foxp3* expression, with ∼15% of cells expressing *Il4^GFP^* in cultures containing IL-4 and IL-2 but not IL-2 alone (Fig. 5, B and C). Thus, IL-4 was sufficient to destabilize *Foxp3* expression and promote redifferentiation of T reg cells into ex-Foxp3 Th2 cells. Titration of IL-4 identified that microRNA 182 (miR-182), which is elevated in T reg cells in Th2-rich environments (Kelada et al., 2013), and GATA-3, previously associated with a subset of intestinal T reg cells (Wohlfert et al., 2011), were both increased upon stimulation with IL-4 (Fig. 5, F and G). Of note, concentrations of IL-4 at 2.5 ng/ml, similar to those required for the polarization of nT cells to Th2 cells (Fig. 5 D), could promote instability of *Foxp3* and acquisition of *Il4^GFP^* by HpTR cells in vitro (Fig. 5 E) but did not regulate *mir182* or *Gata3* in HpTR cells. Only at higher concentrations of IL-4 (≥5 ng/ml) were *mir182* and *Gata3* regulated, suggesting that they may reinforce a Th2 cell lineage in a population of ex-Foxp3 cells.

### Ex-Foxp3 Th2 cells develop in an IL-4Rα–dependent manner in vivo

**Figure 6.**
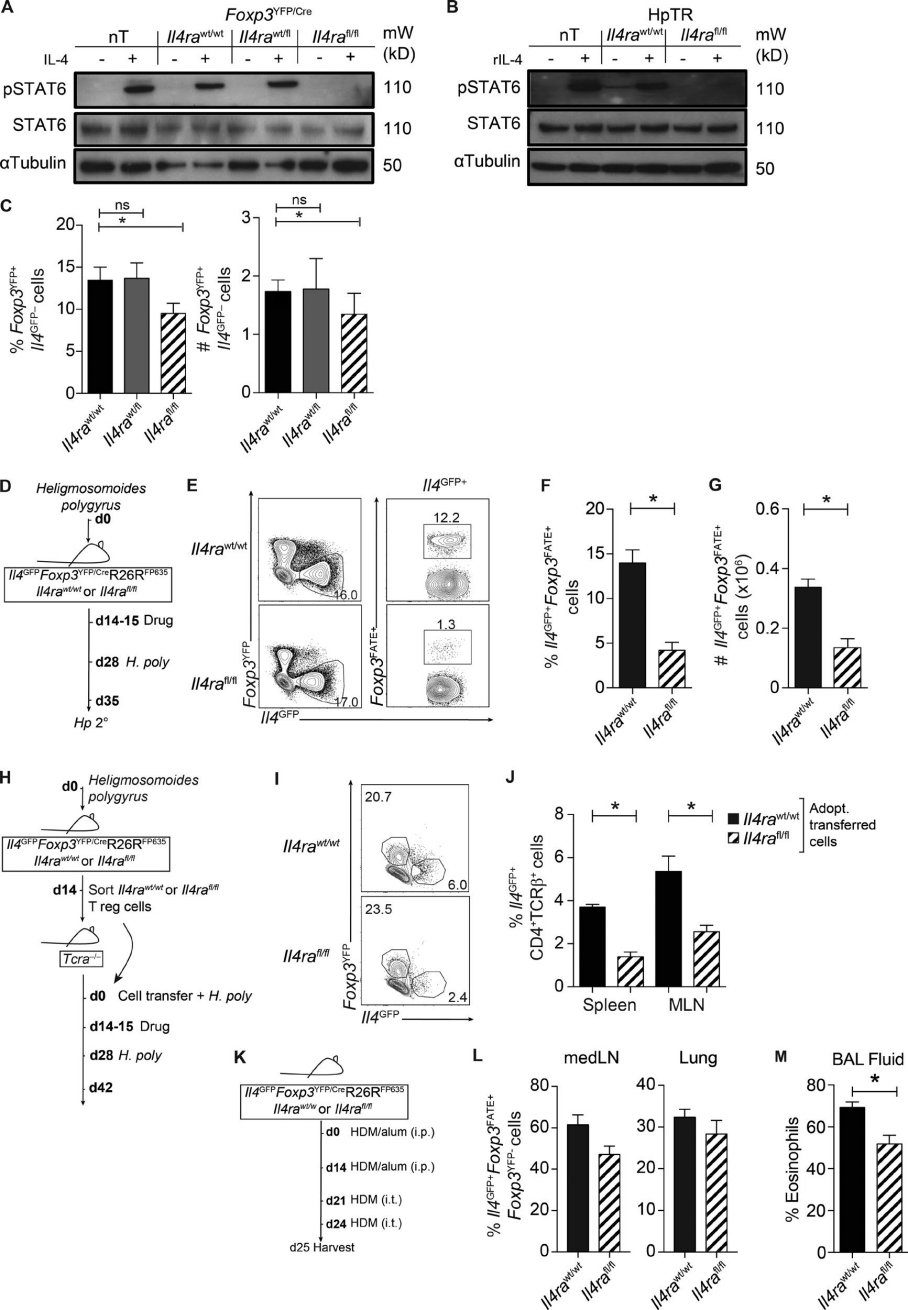
**IL-4 signaling in T reg cells is required for the development of ex-Foxp3 Th2 cells in vivo.** (A and B) nT, *Il4ra^fl/fl^*, *Il4ra^fl/wt^*, or *Il4ra^wt/wt^* HpTR cells were sort purified from naive (A) or *Hp* 1° (B) *Il4ra^wt/wt^* or *Il4ra^fl/fl^* fate-reporter mice day 14 after infection and stimulated with recombinant IL-4 at 37°C for 15 min or media as a control. Levels of pSTAT6, total STAT6, and α-tubulin protein in restimulated cells are shown. Data are representative of two independent experiments. Sorted T cells were pooled from two to three mice. mW, molecular weight. (C) Proportion and absolute number of *Foxp3^YFP+^* cells in the spleen of naive *Il4ra^fl/fl^*, *Il4ra^fl/wt^*, or *Il4ra^wt/wt^* fate-reporter mice. Data are representative of two independent experiments. (D) Experimental model. *Il4^GFP^Foxp3^YFP/Cre^*R26R^FP635^Il4ra^fl/fl^ mice were infected with 200 *H. polygyrus* larvae. *Hp* 2° mice were treated with pyrantel embonate on day 14–15 and reinfected on day 28. Mice were harvested at day 7 after infection. (E) Representative FACS plots of *Foxp3^FATE^* expression within CD4^+^TCRβ^+^*Il4^GFP+^* cells in *Il4ra^fl/fl^* or *Il4ra^wt/wt^ Hp* 2° mice day 7 after infection. (F and G) Proportion (F) and absolute number (G) of CD4^+^TCRβ^+^*Il4^GFP+^Foxp3^FATE+^* cells in the spleen. Data represent two independent experiments with three to four mice per group. (H) Experimental model. T reg cells were sort purified from *Il4ra^wt/wt^* or *Il4ra^fl/fl^ Hp* 1° mice day 14 after infection. *Il4ra^wt/wt^* or *Il4ra^fl/fl^* T reg cells were transferred to *Tcra^−/−^* mice. Recipient mice were infected with *H. polygyrus*, treated with pyrantel embonate at days 14–15, and then infected with *H. polygyrus* at day 35 and harvested at day 42 after transfer (see experimental model in Fig. 2 A). (I and J) Representative FACS plots of CD4^+^TCRβ^+^*Il4^GFP+^* cells (I) and frequency of *Il4^GFP+^* cells (J) in the spleen and MLN of *Il4ra^fl/fl^* or *Il4ra^wt/wt^* HpTR-recipient mice day 14 after infection. Data are representative of two experiments with three to five mice per group. Adopt., adoptively. (K) Experimental model. In brief, *Il4ra^fl/fl^* or *Il4ra^wt/wt^* mice were subjected to a model of HDM/alum sensitization. i.t., intratracheally. (L) Frequency of CD4^+^TCRβ^+^*Il4^GFP+^Foxp3^FATE+^* cells in mediastinal LNs (medLN) and lungs 24 h after challenge. (M) Proportion of eosinophils in the BAL fluid. Data represent two independent experiments with four to nine mice per group. *, P ≤ 0.05; Mann-Whitney test. Error bars represent SEM.

After the observation that IL-4 was sufficient to reprogram T reg cells into ex-Foxp3 Th2 cells, we tested whether IL-4 was necessary for their development in vivo. We crossed fate-reporter mice (*Il4^GFP^Foxp3^YFP/Cre^*R26R^FP635^) with *Il4ra^fl/fl^* mice to generate mice with a conditional deletion of IL-4Rα on *Foxp3*-expressing cells (*Il4^GFP^Foxp3^YFP/Cre^*R26R^FP635^*Il4ra^fl/fl^*) and track the ontogeny of Th2 cells. T reg cells isolated from *Il4ra^fl/fl^* mice were unresponsive to IL-4 signaling in vitro compared with T reg cells from both *Il4ra^wt/wt^* or *Il4ra^fl/wt^* mice (as measured by pSTAT6; Fig. 6 A), confirming that IL-4–signaling was abrogated in *Foxp3*-expressing cells. Furthermore, *Foxp3^YFP+^* cells isolated from *H. polygyrus*–infected mice (HpTR) had reduced levels of pSTAT6 ex vivo (Fig. 6 B), further highlighting a role for IL-4 signaling in T reg cells during infection (see Fig. 5 A). Of note, the proportion and absolute number of *Foxp3^YFP+^* cells was slightly reduced in *Il4ra^fl/fl^* mice compared with *Il4ra^wt/wt^* mice (Fig. 6 C), suggesting that some T reg precursors may require IL-4.

After secondary infection of *Il4ra^fl/fl^* mice (Fig. 6 D), the proportion and absolute number of ex-Foxp3 Th2 cells were significantly reduced compared with *Hp* 2° *Il4ra^wt/wt^* mice (Fig. 6, E–G), demonstrating a fundamental requirement for IL-4 signaling in *Foxp3*-expressing cells for the development of ex-Foxp3 Th2 cells. To further test whether IL-4 was required for CD25^high^ T reg cells to convert to Th2 cells, we adoptively transferred sort-purified *Foxp3^YFP+^Foxp3^FATE+^*CD25^high^ cells (with a demethylated *Foxp3* locus; Fig. 4 I) to *Tcra^−/−^* mice and subjected recipient mice to a secondary *H. polygyrus* infection (Fig. 6 H), as previously described in Fig. 2. The proportion of *Il4ra^fl/fl^* T reg cells converting to *Il4^GFP+^* cells was significantly reduced after adoptive transfer (Fig. 6, I and J), again indicating that IL-4Rα signaling was essential for the conversion of T reg cells to Th2 cells.

To determine whether the development of ex-Foxp3 Th2 cells was restricted to antihelminth immune responses in the intestine, we measured the frequency of ex-Foxp3 Th2 cells in the lung and local draining mediastinal LN after HDM-induced airway allergy (Fig. 6 K). In the mediastinal LN and lung, we observed that up to 60% and 30%, respectively, of Th2 cells had originated from ex-Foxp3–expressing cells. Unlike responses in the small intestine, the absence of *Il4ra* on T reg cells only modestly reduced the frequency of ex-Foxp3 Th2 cells (Fig. 6 L), suggesting that IL-4R–independent mechanisms also contribute to T reg cell conversion after acute HDM-driven airway inflammation. However, the modest reduction of ex-Foxp3 Th2 cells in the absence of *Il4ra*-expressing T reg cells led to a significant reduction of airway eosinophils (Fig. 6 M), suggesting that *Il4ra*-dependent ex-Foxp3 Th2 cells contribute HDM-driven airway eosinophilia.

### Ex-Foxp3 Th2 cells exhibit Th2 effector functions and are sufficient to promote immunity to *H. polygyrus*

**Figure 7.**
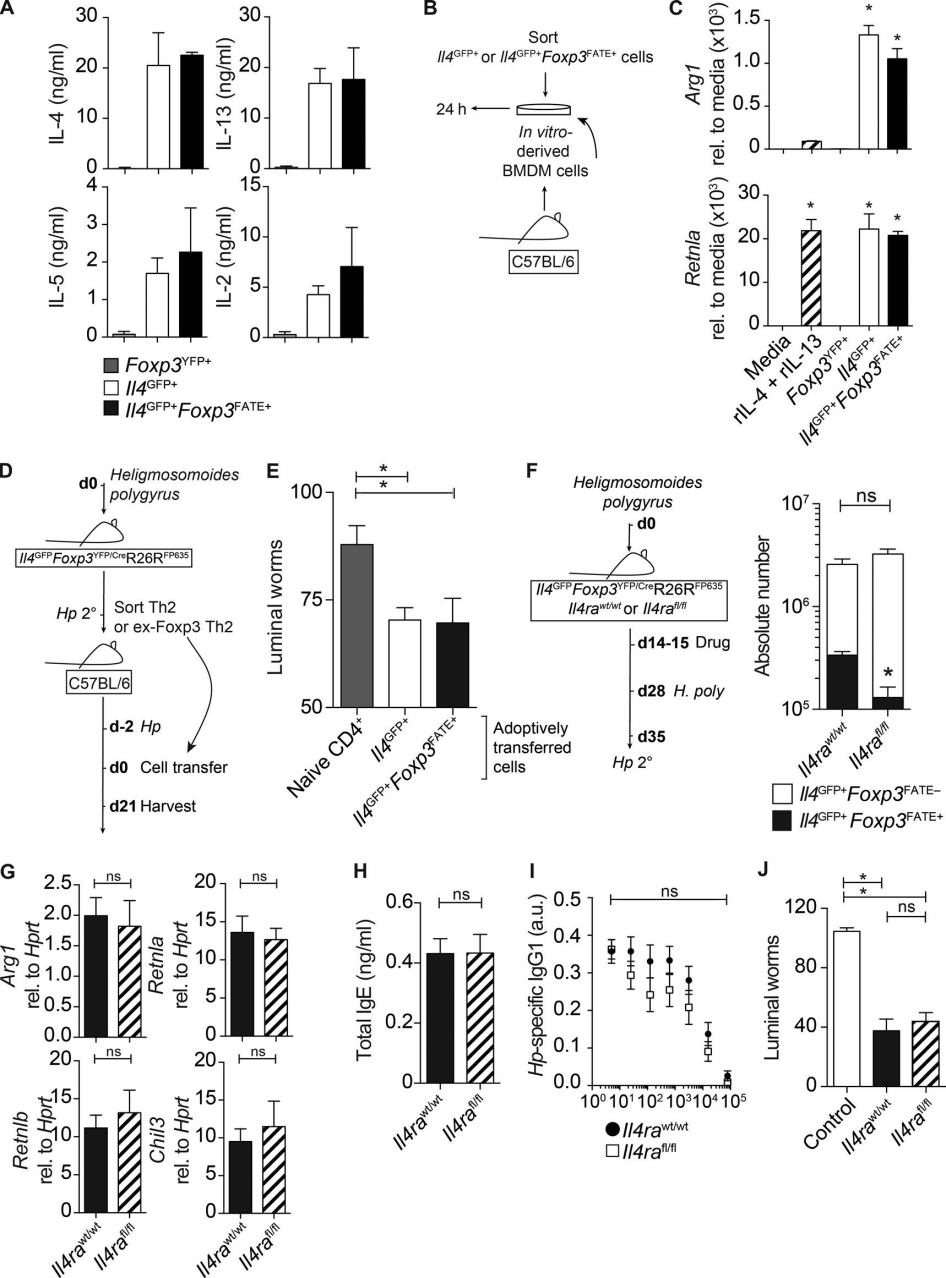
**Ex-Foxp3 Th2 cells secrete type-2 cytokines, promote AAMφ in vitro, and are sufficient to drive the expulsion of *H. polygyrus*.** (A–J) T reg, Th2, and ex-Foxp3 Th2 cells were sort purified from *Hp* 1° and *Hp* 2° *Il4^GFP^Foxp3^YFP/Cre^R26R^FP635^* mice and stimulated with PMA/ionomycin for 24 h. (A) Concentration of IL-4, IL-13, IL-5, and IL-2 in the supernatant of restimulated cells. Three technical replicates were used. (B) BMDMs were cultured with FACS-purified T cells for 24 h or with media and recombinant IL-4 + IL-13. (C) Expression of *Arg1* and *Retnla* in stimulated BMDMs. Data are representative of two to three independent experiments with three technical replicates. Sort-purified T cells were pooled from three to four donor mice. rel., relative. (D) Th2 and ex-Foxp3 Th2 cells were sort purified from *Hp* 2° *Il4^GFP^Foxp3^YFP/Cre^*R26R^FP635^ mice at day 14 after infection and transferred to *Hp* 1°C57BL/6 recipients 2 d after infection. (E) Intestinal worm burden at day 21 after infection. Data represent two pooled experiments with 6–10 mice per group. (F) Experimental model (see model in Fig. 6 D). Absolute number of CD4^+^TCRβ^+^*Il4^GFP+^Foxp3^FATE–^* and CD4^+^TCRβ^+^*Il4^GFP+^Foxp3^FATE+^* cells in the spleens is shown. Four mice per group were used. (G) Small intestine expression of key type-2 response genes, expressed relative (rel.) to *Hprt*. Four mice per group were used. (H and I) IgE (H)- and *H. polygyrus* (I)–specific IgG1 levels in the serum. Data represent two independent experiments with three to four mice per group. a.u., arbitrary units. (J) Intestinal worm count day 14 after infection. Data represent two independent experiments with five to seven mice per group. *, P ≤ 0.05; Mann-Whitney test. Error bars represent SEM.

In accordance with our observations that ex-Foxp3 Th2 cells had largely lost their T reg cell phenotype, purified ex-Foxp3 Th2 cells secreted high concentrations of characteristic Th2 cytokines IL-4, IL-5, IL-13, and IL-2, similar to Th2 cells (Fig. 7 A). AAMφ is dependent on IL-4 and IL-13 (Gordon and Martinez, 2010) and is required for expulsion of *H. polygyrus* (Anthony et al., 2006). Therefore, we tested whether ex-Foxp3 Th2 cells could alternatively activate BMDMs in vitro (Fig. 7 B). Indeed, co-culturing ex-Foxp3 Th2 cells with BMDMs led to high expression of the characteristic markers *Arg1* and *Retnla* in BMDMs (Fig. 7 C), suggesting that ex-Foxp3 Th2 cells may function in vivo during memory responses to *H. polygyrus*. To test whether ex-Foxp3 Th2 cells could promote immunity to *H. polygyrus*, we transferred ex-Foxp3 Th2 cells or conventional Th2 cells isolated from *Hp* 2° mice to WT *Hp* 1° mice and assessed luminal worm burdens 21 d after infection (Fig. 7 D). Both Th2 and ex-Foxp3 Th2 cells were able to passively transfer immunity to normally susceptible *Hp* 1° hosts, resulting in a significant reduction in the establishment of infection, compared with mice receiving naive CD4^+^ T cells (Fig. 7 E). Thus, ex-Foxp3 Th2 cells were functionally indistinguishable from conventional Th2 cells in vitro and in vivo and were sufficient to drive immunity to *H. polygyrus*.

Although ex-Foxp3 Th2 cells were significantly impaired in *Il4ra^fl/fl^* mice (Fig. 6, F and G), the total number of *Il4^GFP+^* Th2 cells was not changed, suggesting dynamic immune compensatory mechanisms fulfilled the net Th2 cell requirement in the context of a whole mouse (Fig. 7 F). Consequently, the activation of downstream Th2 cell–driven mechanisms including AAMφ (Fig. 7 G) and B cell class switching to IgE- and IgG1-secreting cells (Fig. 7, H and I) and subsequent immunity to secondary *H. polygyrus* infection were preserved in *Hp* 2° *Il4ra^fl/fl^* mice (Fig. 7 J).

In conclusion, during Th2 cell–dependent immunity to *H. polygyrus*, a significant proportion of Th2 cells develop from Foxp3^+^ cells in an IL-4–dependent manner. Furthermore, ex-Foxp3 Th2 cells were sufficient to activate innate effector pathways and promote expulsion of *H. polygyrus*. Collectively, this study identifies a previously unappreciated origin of Th2 cells after helminth infections and suggests that targeting IL-4R signaling on T reg cells may promote Th2 cells, simultaneously curbing T reg cells, to enhance antihelminth immunity.

## Discussion

The balance between T reg and effector T cells can determine the establishment, chronicity, and severity of infection. In extreme cases, acute loss of T reg cells can be lethal (Oldenhove et al., 2009), whereas expansion of T reg cells can permit chronic infection (Finney et al., 2007; Rausch et al., 2009; Grainger et al., 2010). It has recently emerged that T reg cells are heterogeneous, with T reg cell–mediated immune homeostasis requiring a degree of specialization for migration to unique environments and for targeting distinct immune cell subsets (Campbell and Koch, 2011). Specifically, coexpression of the transcription factors *Irf4* and *Foxp3* is required for T reg cells to prevent pathogenic type-2 inflammation (Zheng et al., 2009). IRF4 is also required for Th2 cell differentiation (Rengarajan et al., 2002), suggesting that factors that influence effector T cell differentiation may also guide T reg cell specialization, in this case IL-4–induced IRF4 (Rengarajan et al., 2002).

T reg cells and Th2 cells may be closely related, with several studies suggesting that loss of *Foxp3* expression correlates with the acquisition of Th2 cell–like phenotype (Wang et al., 2010; Hansmann et al., 2012). Despite such evidence for a functional relationship between T reg and Th2 cells, the development and function of IL-4–secreting ex-Foxp3 cells has not been characterized. In this study, we investigated the relationship between T reg and Th2 cells during an infection with the intestinal helminth *H. polygyrus* and after HDM-induced airway allergy and observed that type-2 immunity involved the expansion of a functional population of ex-Foxp3 Th2 cells with varying degrees of dependency on IL-4Rα expression on T reg cells.

In both gain- and loss-of-function systems, it has long been appreciated that IL-4 is essential for protective immunity to *H. polygyrus* (Urban et al., 1991b, 1995; Finkelman et al., 1997; Herbert et al., 2009). However, the essential source and targets of IL-4 have remained elusive. Supraphysiological levels of IL-4 can promote expulsion of a naturally chronic *H. polygyrus* infection (Urban et al., 1991b), and the adoptive transfer of Th2 cells, in this study and others, was sufficient to promote expulsion of *H. polygyrus* infection. These observations led to the notion that T cells were important targets of IL-4. Indeed, depletion of T cells abrogated immunity (Urban et al., 1991a), further supporting the view that CD4^+^ T cells are critical for immunity. In this study, we found that T reg cells were actively phosphorylating STAT6 during infection and that IL-4R signaling was required for the conversion of T reg cells to Th2 cells in vitro and in vivo. Thus, a role for CD4^+^ T reg cells as a target of IL-4 may have been overlooked in these previous studies.

The secretion of IL-2, IL-4, IL-5, and IL-13 by ex-Foxp3 Th2 cells at similar levels as conventional Th2 cells suggested that ex-Foxp3 Th2 cells could function as Th2 effector cells. Indeed, in vitro and in vivo, ex-Foxp3 Th2 cells could alternatively activate macrophages and mediate *H. polygyrus* expulsion, similar to Th2 cells. Transcriptional analysis of converted T reg cells that had up-regulated *Il4* (Fig. 2) identified that T reg cells had significantly changed their transcriptional profile, more closely resembling Th2 cells than their T reg cell past and also that they differentially expressed >500 unique genes distinct from their T reg cell past and from Th2 cells.

It has been widely reported that T reg cells have reduced TCR activity relative to effector T cells (for review, see Campbell and Ziegler, 2007). Notably, in our datasets, T reg cells that had up-regulated *Il4* also up-regulated several genes involved in TCR signaling (Table S2). For example, we observed elevated expression levels of Syk, Zap70, Jak3, inositol polyphosphate phosphatase-like 1 (*Inppl1*; *Ship2*), and protein tyrosine phosphatase nonreceptor type 22 (*Ptpn22*). Furthermore, Ship1 (Collazo et al., 2012) and Ptpn22 (Nowakowska and Kissler, 2016) negatively regulate T reg cell development, supporting the hypothesis that elevated TCR activity may contribute to the conversion of T reg cells into effector T cells. Many of the differentially regulated genes were distinct from conventional Th2 cells, suggesting that converted T reg cells may also have unique properties. At least one of these properties was their ability to reexpress *Foxp3* after exposure to TGFβ (Fig. 4, K and L), unlike conventional Th2 cells. Whether these cells represent a population of highly plastic, TGFβ-responsive cells that retain a demethylated *Foxp3* locus is currently unclear.

Mechanistically, it has previously been demonstrated that IL-6 and IL-4 signaling (Kastner et al., 2010) through STAT6 antagonizes the demethylation of the *Foxp3* locus and destabilizes T reg cells (Wei et al., 2007; Feng et al., 2014). In this study, we also found that IL-4–dependent ex-Foxp3 Th2 cells had a methylated *Foxp3* locus, supporting the hypothesis that IL-4 signaling contributes to the remethylation of the *Foxp3* locus in T reg cells. These observations are in line with a previous study using *Il4ra*-transgenic T reg cells, which have supraphysiological IL-4R–mediated signaling and acquired a Th2 effector profile (Noval Rivas et al., 2015). We have previously identified that IL-4 selectively up-regulated miR-182 in T reg cells to limit *Bach2* expression and IL-2 secretion, maintaining T reg cell–mediated control of type-2 inflammation (Kelada et al., 2013). Collectively with this study, we hypothesized that an IL-4R–signaling gradient fine-tunes T reg cells to control Th2 responses before converting T reg cells into Th2 cells, as we show here. However, increasing concentrations of IL-4 led to an increasing percentage of T reg cells losing *Foxp3* expression and up-regulating IL-4, and only at higher doses of IL-4 did we observe increased miR-182 and *Gata3* expression in T reg cells. Single-cell analysis is required to determine whether dose-dependent IL-4R signaling destabilizes Foxp3 before Gata3 activation and up-regulation of IL-4 in T reg cells or whether IL-4 primes a distinct population of T reg cells for an appropriate Th2 regulatory program. Nevertheless, these data suggest that there is a graded response to IL-4 in T reg cells and that additional factors most likely contribute to T reg cell conversion, including TCR signal strength, duration of IL-4 exposure, and appropriate co-stimulation and metabolic reprograming.

It has previously been reported that activation of human T cells can lead to the transient expression of *Foxp3* (Wang et al., 2007). If the same occurred in mice, our fate-marking system would mark all activated cells. The observation that 10–20% of Th2 cells were fate marked, and not all activated Th2 cells, led us to the conclusion that *Foxp3* was not transiently expressed in activated mouse T cells. This is also supported by the fact that ex-Foxp3 Th2 cells were significantly reduced in *Il4ra^fl/fl^* mice. Despite up to 20% of Th2 cells originating from Foxp3^+^ cells, deletion of *Il4ra* on T reg cells, which led to a significant reduction of ex-Foxp3 Th2 cells, did not impact the total number or frequency of Th2 cells. This was because of an increase in the number of conventional Th2 cells, compensating for the loss of ex-Foxp3 Th2 cells. This dynamic compensation ensured that sufficient Th2 cells were generated to activate innate cells and class-switch B cells and mediate parasite expulsion.

In summary, we have identified a previously unappreciated and intimate relationship between T reg cells and effector Th2 cells during intestinal helminth infections. Targeting immunoregulatory pathways may temporarily curtail overly regulated responses to increase immunity in the face of chronic infections.

## Materials and methods

### Animals

All mice (C57BL/6, *Tcra^−/−^* [Mombaerts et al., 1992], 4get [Mohrs et al., 2001], *FILIG* [Wan and Flavell, 2007], *Foxp3^YFP/Cre^* [Rubtsov et al., 2008; provided by A.Y. Rudensky, Memorial Sloane Kettering Cancer Center, New York, NY], *Il4ra^fl/fl^* [Herbert et al., 2004], and *R26R^FP635^* [Coomes et al., 2015) were maintained under specific pathogen–free conditions at the Mill Hill Laboratory, The Francis Crick Institute, on a C57BL/6 background. *Il4ra^fl/fl^* mice (Herbert et al., 2004) were originally backcrossed onto a C57BL/6 background at The University of Edinburgh by Dominik Ruckerl and Judith E. Allen. All animal experiments were approved by The Francis Crick Institute Ethical Review Panel and UK Home Office regulations (project licenses 80/2506 and 70/8809).

### *H. polygyrus* infection

Mice were infected with 200 *H. polygyrus* L3 larvae by oral gavage. For some experiments, mice were treated with 2.5 mg/ml of the anthelmintic pyrantel embonate (Pfizer) on days 14 and 15 and subsequently given a challenge infection with 200 *H. polygyrus* L3 larvae on day 28. Mice were harvested at day 7, 14, or 23 after infection, as indicated. Adult worms were counted in the lumen of the intestine on day 14 or 23 after primary or secondary infection using a stereoscopic microscope (SMZ-2B; Nikon).

### HDM-induced airway inflammation

Mice were sensitized intraperitoneally twice with 100 µg (dry weight) HDM (*Dermatophagoides pteronyssinus* extracts; Greer) with Imject Alum (Thermo Fisher Scientific) diluted in PBS (1:3) solution. After sensitization, mice were challenged twice intratracheally with 100 µg HDM on days 21 and 24. All the parameters for airway allergy were measured 24 h after the last challenge.

### Bronchoalveolar lavage (BAL) fluid preparation and differential cell counts

1 d after the last HDM challenge, mice were culled, and BAL fluid was collected using 1.5 ml PBS for each mouse. The total number of BAL cells was counted, and differential cell counts were performed on cytospin preparations stained with Giemsa stain (modified; Sigma-Aldrich).

### Cell isolation, RNA extraction, and qRT-PCR

Spleen, MLN, and PP cells were made into single-cell suspensions in complete IMDM and prepared for FACS analysis or sorting. Red blood cells were lysed using ACK lysis buffer (Thermo Fisher Scientific). For qRT-PCR, purified cells and tissue were harvested in RLT lysis buffer (QIAGEN) or RNAlater (Thermo Fisher Scientific). Tissues were homogenized, RNA was extracted, and cDNA was generated as previously described (Pelly et al., 2016) or with miSCRIPT II and HiFlex buffer (QIAGEN) for miRNA expression analysis. cDNA was amplified and normalized to the house-keeping gene *Hprt* or *rnu6b* (Invitrogen) for miRNAs and expressed as fold-change (as indicated in the figure legends). The sequences for primers used are listed in Table 1 or are previously published (Pelly et al., 2016). miRNA primers were obtained from QIAGEN.

**Table 1.** Real-time PCR primers

	Primers	
*Foxp3*	GTGGGCACGAAGGCA AAG	CCTTGTTTTGCGCTGAGAGTCT

### Flow cytometry and FACS sorting

Cell sorting was performed using a FACSAria II (BD) or Influx (BD) flow cytometer. Single-cell suspensions were stained with antibodies in PBS containing 2% FBS for 25 min at 4°C and sorted in phenol red–free complete IMDM containing 1% FBS and 1 mM EDTA. FACS analysis was performed using either an LSRII or LSRFortessa X-20 flow cytometer (BD). For FACS analysis, stained cells were fixed in 4% formaldehyde (Sigma-Aldrich) for 20 min at 4°C. The viability of cells was determined using a LIVE/DEAD Fixable Blue kit (Thermo Fisher Scientific). Intracellular cytokine staining was on 0.05 mg/ml PMA (Promega)– and 0.1 mg/ml ionomycin (Sigma-Aldrich)–stimulated cells in the presence of GolgiStop (BD) and GolgiPlug (BD) for 6 h at 37°C. Cells were permeabilized for 30 min at 4°C followed by staining in permeabilization buffer (eBioscience). The antibodies used include: CD4 (RM4-5: eFluor 450 [eBioscience] and APC [BioLegend]; and MCD0430: Pacific orange [Invitrogen]), TCRαβ (H57-597: APC [eBioscience], PeCy7, and PerCPCy5.5 [BioLegend]), CD25 (PC61: APC [eBioscience], APCCy7 [BioLegend], and PerCPCy5.5 [eBioscience]), CD44 (IM7: PeCy7 [BioLegend] and PERCPCy5.5 [eBioscience]), IL-4 (PE; eBioscience), IL-5 (554396: APC; BD), IL-13 (eBio13A; FITC; eBioscience), Foxp3 (FJK-16s: PE and APC; eBioscience), CTLA-4 (UC10-4F10-11: APC; BD), CD103 (2E7: APC; eBioscience), and GITR (DTA-1: eFluor450; eBioscience).

### Adoptive cell transfer model

Male mice were infected with 200 *H. polygyrus* larvae for 14 d. CD4^+^TCRβ^+^*Foxp3^RFP+^Il4^GFP–^*CD25^high^ (HpTR) cells were sort purified from CD4-enriched spleens and MLNs (Miltenyi Biotec) of *H. polygyrus*–infected reporter mice day 14 after infection. In some experiments, cells were sort purified as CD4^+^TCRβ^+^*Foxp3^RFP+^Il4^GFP−^*CD25^low^ (as specified in the figure legends). CD4^+^TCRβ^+^*Foxp3^RFP–^Il4^GFP–^*CD25^–^CD44^low^ nT cells were sorted from naive double-reporter mice. Sort-purified T cells were counted in Trypan blue (Sigma-Aldrich) using a cell-counting hemocytometer (Hawksley) and an LED inverted light microscope (Leica Biosystems) and diluted in sterile PBS for i.v. delivery. For each experiment, 0.5–1.5 × 10^6^ HpTR or nT cells were injected into male *Tcra^−/−^* mice infected with 200 *H. polygyrus* larvae on the day of transfer. Recipient mice were drug cured and reinfected with *H. polygyrus* (see model in Fig. 2 A).

### Th cell polarization

10^5^ sort-purified HpTR or nT cells were plated onto tissue culture–treated flat-bottom 96-well plates that were coated with 1 µg/ml CD3 (Bio X Cell) and 10 µg/ml CD28 (Bio X Cell) antibody at 37°C for 2–3 h. For Th2 cell polarizations, cells were cultured with recombinant IL-2 (10 ng/ml; R&D Systems) and/or IL-4 (10 ng/ml or otherwise indicated in Fig. 5; PeproTech). For T reg cell polarizations, cells were cultured with 5 ng/ml recombinant TGFβ (Insight Biotechnology) and 5 ng/ml IL-2 (R&D Systems). At day 3, cells were removed from the plate and transferred to a round-bottom plate and left for an additional 4 d. Cells were harvested at day 7 for FACS analysis.

### BMDM culture and co-culture with T cells

BM cells were flushed from the femur of male C57BL/6 mice, filtered through a 40-µM filter, and centrifuged at 1,500 rpm for 5 min. Cells were lysed in ACK lysis buffer (Thermo Fisher Scientific; 2 ml per mouse for 1.5 min), washed, and centrifuged at 1,500 rpm for 5 min. 5 × 10^6^ cells were plated in Petri dishes in conditioned media (Dulbecco’s Modified Eagle Medium with GlutaMAX; Thermo Fisher Scientific), 20% L-cell 929 (in-house media kitchen; Mill Hill Laboratory), 10% FBS, 10 mM Hepes, 100 U/ml penicillin and 100 µg/ml streptomycin (Gibco), 2.7 mM l-glutamine (Gibco), 0.05 mM 2-mercaptoethanol (Gibco), and 1 mM sodium pyruvate (Lonza). Additional conditioned media was added at day 4. Adherent cells were harvested at day 7 and resuspended in 1% DMEM with GlutaMAX (Thermo Fisher Scientific), 1% FBS, 10 mM Hepes, 100 U/ml penicillin and 100 µg/ml streptomycin (Gibco), 2.7 mM l-glutamine (Gibco), 0.05 mM 2-mercaptoethanol (Gibco), and 1 mM sodium pyruvate (Lonza). 10^6^ BMDMs were plated in 24-well flat-bottom tissue culture–treated plates and left to rest for 24 h. 10^5^ sort-purified T cells were resuspended in 1% DMEM containing 1 µg/ml soluble CD3 and 10 µg/ml soluble CD28 antibody (Bio X Cell) and cultured with the BMDMs for 24 h. As a control, BMDMs were co-cultured in the presence of 20 ng/ml recombinant IL-4 (PeproTech) and 20 ng/ml IL-13 (PeproTech) or media alone. Nonadherent cells were washed off after 24 h, and adherent activated BMDMs were harvested for downstream analysis.

### ELISAs and cytokine measurements

Cytokine concentrations were measured in cell culture supernatants using either FlowCytomix (eBioscience) or a LegendPlex Mouse Th1/Th2 Panel (BioLegend) flow cytometry multi-analyte detection system for IL-4, IL-2, IL-5, and IL-13 per the manufacturer’s instructions. Serum IgE (and purified mouse IgE standard; BD) was captured overnight on a plate coated with 2 µg/ml rat anti–mouse IgE (R35-72; BD) and detected with biotin rat anti–mouse IgE at 1 µg/ml (R35-118; BD), streptavidin HRP (BD), and ABTS One Component HRP Microwell substrate (SurModics). *H. polygyrus* antigen (HEX) was obtained by homogenizing adult worms in PBS. Serum antigen-specific IgG1 was captured on a plate coated with 5 µg/ml HEX and detected using biotin rat anti–mouse IgG1 (Invitrogen), streptavidin HRP, and ABTS.

### Western blotting

For immunoblotting, cells were lysed in 1× radioimmunoprecipitation assay buffer (500 mM Tris HCl, pH 2.5, 150 mM NaCl, 2 mM EDTA, 0.1% SDS, 0.5% deoxycholate, and 1% Nonidet-P40) containing protein inhibitors as per the manufacturer’s instructions (diluted 1:50; Roche), 5 mM NaF, 1 mM Na_3_VO_4_, 100 nM okadoic acid, 2 mM Na_4_P_2_O_7,_ and MilliQ water. Cell lysates were normalized to equal total protein content using a BCA Protein Assay kit (Thermo Fisher Scientific) and resolved on 10% Criterion TGX Gels (Bio-Rad Laboratories). Separated proteins were transferred onto Trans-Blot Turbo polyvinylidene fluoride transfer (Bio-Rad Laboratories) membranes. Membranes were blocked in 0.1% PBS-Tween (PBST; Sigma-Aldrich) containing 20% milk (Sigma-Aldrich) and then incubated with primary (pSTAT6 and STAT6, Cell Signaling Technology; α-tubulin, in house) and secondary (rabbit IgG; GE Healthcare) antibodies in 0.1% PBST (Sigma-Aldrich) containing 10% milk (Sigma-Aldrich). Membranes were washed in PBST, and specific bound antibodies were visualized by chemiluminescence (Immobilon; EMD Millipore).

### Microarray analysis

HpTR and nT cells were sort purified from CD4-enriched spleens and MLNs of *H. polygyrus*–infected reporter mice day 14 after infection, as described in the Adoptive cell transfer model section. CD4^+^TCRβ^+^*Il4^GFP+^Foxp3^RFP–^* (HpTR→*Il4^GFP+^*) were sort purified from HpTR *Tcra^−/−^* recipients and CD4^+^TCRβ^+^*Il4^GFP+^Foxp3^RFP–^* (nT→*Il4^GFP+^*) were sort purified from nT *Tcra^−/−^* recipients day 42 after adoptive transfer. RNA was extracted from the sort-purified populations and concentrated using a MiVac DNA concentrator (Barnstead; Genevac). The Systems Biology Unit at The Francis Crick Institute, Mill Hill Laboratory, processed samples for microarray analysis. RNA quality was determined using a bioanalyzer (2100; Agilent Technologies). RNA concentrations were determined using a Qubit 2.0 Fluorometer (Thermo Fisher Scientific). cDNA was amplified from 20 ng total RNA using the Ovation Pico WTA system (version 2; NuGEN). Amplified cDNA was fragmented and labeled using the Encore Biotin Module (NuGEN). Labeled cDNA was hybridized to a GeneChip mouse Genome 430A 2.0 microarray using the GeneChip Hybridization, Wash, and Stain kit (Affymetrix) and run on the GeneChip Fluidics Station (450; Affymetrix) followed by scanning on a GeneChip Scanner (3000 7G; Affymetrix). Microarray data were analyzed using GeneSpring software (Agilent Technologies). Samples were normalized using the MicroArray Suite 5 method (MAS5; Affymetrix) and filtered by Flags and expression (20–100th percentile). Differentially expressed genes were determined using unpaired Student’s *t* tests relative to nT control cells. Genes with false discovery rate–corrected p-values <0.05 and fold-change values ≥2 were considered significant. Three biological replicates of each sample were used. Each biological replicate contains cells pooled from three to four mice. Three-way comparative analyses and predicted upstream regulators were determined using Ingenuity Pathways Analysis (Ingenuity systems).

### Bisulfite modification of DNA, cloning, and sequencing

HpTR, nT, and HpTR→*Il4^GFP+^* cells were obtained as described in the Adoptive cell transfer model section. CD4^+^TCRβ^+^*Il4^GFP–^Foxp3^YFP+^* (*Foxp3^YFP+^*), CD4^+^TCRβ^+^*Il4^GFP+^Foxp3^YFP–^* (*Il4^GFP+^*), and CD4^+^TCRβ^+^*Il4^GFP+^Foxp3^YFP–^Foxp3^FATE+^* (*Il4^GFP+^FP635^+^*) cells were sort purified from the spleen and MLN of fate-reporter mice day 7 after secondary infection. Sorted cells were centrifuged at 1,500 rpm for 5 min, and cell pellets were digested in 1× Tris and EDTA buffer containing 200 µg/ml proteinase K and 0.5% SDS (Thermo Fisher Scientific) at 55°C for 3–4 h. DNA was extracted using phenol/chloroform (Sigma-Aldrich), centrifuged (13,000 rpm for 10 min), and washed in 100% chloroform (Sigma-Aldrich). The aqueous phase was removed and added to 100% ethanol for DNA precipitation. For large quantities of DNA, the DNA was fished using a glass pipette, washed in 70% ethanol, and eluted in 30–40 µl distilled H_2_O (dH_2_O). DNA concentrations were measured using a Nanodrop 1000 (Thermo Fisher Scientific). For smaller quantities, the DNA was precipitated in 100% ethanol at −20°C for 20 min and then centrifuged at 13,000 rpm for 10 min and eluted in 30–40 µl dH_2_O. DNA concentrations were measured using the Quant-iT PicoGreen dsDNA Assay kit (Thermo Fisher Scientific). Bisulfite modification was performed as described previously (Susan et al., 1994). In brief, 1 µg DNA was denatured in a final concentration of 0.3 M NaOH at 37°C for 15 min. 2 M metabisulfite (Sigma-Aldrich) and 100 mM hydroquinone (Sigma-Aldrich) were made up in dH_2_O, and the pH was adjusted to 4.5–5. Denatured DNA was modified in bisulfite (6.14 × volume of DNA) containing 0.5 mM hydroquinone. The sample was gently mixed, overlaid with mineral oil, and incubated at 55°C overnight (16–20 h). DNA was recovered from under the mineral oil and purified using a chromatin immunoprecipitation DNA Clean & Concentrator kit (Zymo Research) according to the manufacturer’s instructions and eluted in 50 µl of elution buffer. Freshly prepared NaOH was added to a final concentration of 0.3 M at 37°C for 15 min. The solution was neutralized with 3 M ammonium acetate. DNA was ethanol precipitated and eluted in 20–50 µl dH_2_O. Efficiency of DNA modification was assessed by qRT-PCR with methylation-specific primers (see Table 1) designed on UroGene (Li and Dahiya, 2002). PCR reactions were performed on a MasterCycler Gradient S PCR cycler (Eppendorf) in a final volume containing 1× PCR buffer, 0.5 U Taq polymerase (Thermo Fisher Scientific), 0.4 mM deoxynucleotide triphosphates, 0.2–1 pmol each of forward and reverse primers, and 0.1 µg DNA. The amplification conditions were 94°C for 3 min, 10 cycles of 94°C for 30 s, 63°C down to 53°C for 40 s (touchdown), 72°C for 45 s, 40 cycles of 94°C for 30 s, 53°C for 30 s, 72°C for 45 s, and a final extension step of 72°C for 5 min. Primers used for amplification of bisulfite-modified DNA for sequencing (see Table 2) were designed based on previous studies (Floess et al., 2007) using MethPrimer primer design software (Li and Dahiya, 2002). PCR products were gel purified using a QiaQuick Gel Extraction kit (QIAGEN) according to the manufacturer’s instructions. Purified DNA was concentrated using a speed vacuum concentrator. DNA was cloned into chemically competent *Escherichia coli* (TOP10; OneShot) according to the manufacturer’s instructions, and cells were plated on ampicillin plates (100 mg/L) with 20 mg/ml X-gal. Clones were cultured overnight in Luria-Bertani broth (in-house media kitchen, The Francis Crick Institute, Mill Hill Laboratory) containing 0.1 mg/ml ampicillin (Sigma-Aldrich). 10–16 clones were sent for sequencing (Source Bioscience). Sequencing results were analyzed using Seqbuilder and the online Quantification Tool for Methylation Analysis (QUMA; Kumaki et al., 2008).

**Table 2.** Bisulfite sequencing primers

	Primers	
Bisulfite sequencing		
2a	TGGGTTTTTTTGGTATTTAAGAAAG	AAAAAACAAATAATCTACCCCACAA
2b2	GAAATTTGTGGGGTAGATTATTTGT	AACTAACCAACCAACTTCCTACACTAT
3	TTTTAAGTTTAAAATTAGTTTGGTTAA	CTCAAATCCTTTTTCTATCAAAAATAT
Methylation specific		
Mod	TGATTTTTTTAAAATATAAAGAAATACGG	TCCCAAATACTAAAATCAAAAACATACG
Unmod	TGATTTTTTTAAAATATAAAGAAATATGG	TCCCAAATACTAAAATCAAAAACATACAC
Gen	TGACTCTTCTAAAACACAAAGAAACACGG	TCCCAAGTGCTGGGATCAAAGGCATGCG

### Statistical analysis

Datasets were compared by Mann-Whitney tests or one-way ANOVA using Prism (V.5.0; GraphPad Software). Differences were considered significant at P ≤ 0.05.

### Accession nos.

The microarray data are available in the Gene Expression Omnibus database under accession no. GSE98518.

### Online supplemental material

Tables S1 and S2 are available as Excel files and contain lists of genes from transcriptional analysis of converted HpTR→*Il4^GFP+^* cells and conventional Th2 cells and pathway analysis of differentially expressed genes in HpTR→*Il4^GFP+^* cells and conventional Th2 cells, respectively.

## Supplementary Material

Table S1 (Excel file)

Table S2 (Excel file)
